# Awareness Levels About Specialty Services Offered by the Faculty of Dentistry in Sebha, Libya

**DOI:** 10.7759/cureus.58557

**Published:** 2024-04-18

**Authors:** Sathya Sethuraman, Syed Wali Peeran, Karthikeyan Ramalingam, Aesa Alzaroug Jaber

**Affiliations:** 1 Dentistry and Physiology, Saveetha Dental College and Hospitals, Saveetha Institute of Medical and Technical Sciences, Saveetha University, Chennai, IND; 2 Preventive Dental Sciences, Jazan University, Jazan, SAU; 3 Oral Pathology and Microbiology, Saveetha Dental College and Hospitals, Saveetha Institute of Medical and Technical Sciences, Saveetha University, Chennai, IND; 4 Oral and Maxillofacial Surgery, Faculty of Dentistry, Sebha University, Sebha, LBY; 5 Oral and Maxillofacial Surgery, Faculty of Dentistry, Ajman University, Ajman, ARE

**Keywords:** patients satisfaction, dental-education, dental practice, sebha, southern region, oral health, libya, specialty, dental, awareness

## Abstract

Background: Assessment of awareness levels about any hospital is critical to judge its current standings and plan for future development. Limited literature is available about dental health in Libya.

Aim: This study aimed to study the awareness and perception of the various specialty services offered by the Faculty of Dentistry (SDC), Sebha University for their quality and preference.

Materials and methods: It was a performance monitoring research, and the data was collected by interview using a standardized questionnaire. Non-probability judgmental sampling was used. The offered services included all specialties in dentistry and oral cancer screening. Information relating to the awareness and perception of SDC specialty services was collected with few agree/disagree questions from 450 subjects. Data was collected, tabulated, and analyzed with descriptive statistics using SPSS v23.0 (IBM, Armonk, NY, USA).

Results: About 22.7% (n=102) were in the 45-54 years age group. Self-employed (n=108, 24%) and professional (n=108, 24%) were noted in occupation. Forty-eight (n=216) were graduates. Three hundred twenty-eight respondents (72.9%) were aware about the dental services. Four hundred twenty-eight respondents (95.1%) have visited SDC. Three hundred six respondents (68%) were satisfied, and 66 respondents (14.7%) were very much satisfied. Dental health checkups were predominantly known to the respondents (n=302, 67%). For all the other specialties, the awareness level was low.

Conclusion: The identification of weak areas is crucial for the future planning and development of oral health care rendered by SDC.

## Introduction

Knowledge, attitude, and practice (KAP) surveys are routinely conducted in healthcare. It is representative of the target population to identify what is known, what is believed, and what is done. Information is collected from semi-structured or structured questionnaires and interviews for qualitative and quantitative data [1]. The increasing burden of chronic disease makes improving access to specialty care a top priority in healthcare delivery [2]. Empowered patients could benefit from higher-quality care, cost-effective treatment options, active participation in treatment planning, shorter hospital stays, minimized harm, and upholding dignity. If patient rights are addressed, it could lead to satisfaction, trust, and better healthcare outcomes [3]. Strong doctor-patient relationships are key to quality care and are even more important in the age of health literacy. With ever-increasing access to health information, patients are more informed about their conditions and rights. This necessitates good communication skills, empathy, and ethical conduct from doctors to build trust and navigate shared decision-making [4].

It is performed to assess the public understanding, inform and engage with the public, identify the community needs and barriers, develop public health interventions, and evaluate the effectiveness or impact of such interventions [1]. Researchers have shown that human-centered design can improve prevention efforts of chronic diseases [5]. Deficiencies in healthcare delivery could be based on clinical decision-making, information management, system-level management of patient flow, and quality of care monitoring [2].

Several complex but interrelated factors determine health and well-being. Design thinking applied to questions around health may prove valuable and complement existing approaches. Many public health projects utilizing human-centered design have recently emerged [5]. A designathon is a crowd-sourced activity where a large group generates solutions and increases public awareness about health challenges [6]. Caring for Providers to Improve Patient Experience (CPIPE) intervention has 5 components: provider training, peer support, mentorship, embedded champions, and leadership engagement [7]. Patient insights vary with time and can determine the success of healthcare provided by matching it with their expectations [8]. By promoting patient involvement, patients and professionals will be better prepared to handle the treatment procedures and outcomes.

Libya is a large nation in North Africa bound by Egypt in the east and Algeria in the west. Limited literature about dental health is available in Libya. The Faculty of Dentistry (SDC) is the only government dental college in the Fezzan region of Libya and is under the Ministry of Higher Education and Scientific Research. It has a fully functioning air-conditioned dental clinic that caters to patients of the Fezzan region. SDC regularly advertised the quality of its facilities and best practices provided by the hospital to create awareness and educate the public. However, whether these objectives of improving awareness and attracting more people have been met is unknown, and future activities must be planned accordingly. Hence, this research was initiated to find out the patients' satisfaction level, improve in-house services, and indicate discrepancies within the service delivery system. This research mainly focuses on the behavioral responses of the target population.

There are no previous studies regarding dental services and evaluation of patient satisfaction in Southern Libya. Hence, this study examined public awareness of the Sebha Faculty of Dentistry (SDC) based on its service awareness and public perception. It gauged how familiar was SDC's general multispecialty dental services. It also measured people's overall impression of SDC, including the quality of facilities, services provided, and their general preference for SDC. The study also gathered qualitative feedback to help SDC improve its services and public awareness.

## Materials and methods

The data was collected through an administered questionnaire (Appendix). Ethical clearance for this study was obtained from the Institutional Human Ethical Committee vide Reference Number: IHEC/SDC/FACULTY/22/OPATH/009. The Institutional Ethics Committee functions by the New Drugs and Clinical Trials Rules 2019. The Saveetha Dental College-Institutional Human Ethical Committee (SDC-IHEC) follows procedures that comply with the requirements of the International Conference on Harmonization (ICH) guidance related to Good Clinical Practice (GCP) and applicable Indian and International regulatory regulations.

The questionnaire was designed with structured questions, logical flow, clarity, and simplicity based on Vengadesh and Mohamed [8]. Pre-testing was performed on 25 people in Sebha's Gurdha area, based on open-ended questions which were again proofread, content validated, and retested after 10 days for reliability. The questionnaire was thoroughly pretested to ensure that it captured the right information and produced reliable data.

The researchers collected data from households in Sebha. Four hundred fifty participants were interviewed after getting informed consent. Inclusion criteria were adults greater than 18 years of age who could understand English. If they were unable to read or write in English, their responses were made by family members. Both genders were included in the study. Exclusion criteria were children and adolescents less than 18 years of age, participants who could not understand English, and those who refused to sign the informed consent.

Details about their age, gender, occupation, education level, awareness about dental services in the Faculty of Dentistry, Sebha University (SDC), visit history to SDC, and purpose of visit to SDC were recorded. Their level of satisfaction and agreement was recorded for the quality of dental care provided with international standards. They were asked whether SDC would be their future preferred choice and whether they would recommend SDC to others. The dental facilities were enquired about oral diagnosis, oral surgery, tooth replacement, gum health, caries treatment, and dental health. An open-ended question was also asked to describe their comments or views on improving the services offered by SDC.

The collected data was checked for errors and omissions. Missing entries and wrong entries were rectified by re-interviewing the same respondents. The data was then analyzed to understand people's awareness of and perceptions about SDC. Frequency tables were created to count occurrences in different categories. The variables were described based on the frequency tables. Descriptive statistics were analyzed with SPSS v23.0 (IBM, Armonk, NY, USA).

## Results

This study assessed the awareness and perception of SDC among the population of Sebha, Libya. A total of 450 respondents were interviewed. There were 238 males (52.9%) and 212 females (47.1%) (Table 1).

**Table 1:** Table showing the gender distribution of the respondents

S.No	Gender	Number of Respondents	Percentage
1	Male	238	52.9%
2	Female	212	47.1%
	Total	450	100%

The respondents were grouped as per their age as under 25 years (n=38, 8.5%), 25-34 years (n=92, 20.4%), 35-44 years (n=86, 19.1%), 45-54 years (n=102, 22.7%), 55-64 years (n=76, 16.9%), and beyond 64 years (n=56, 12.4%) (Table 2).

**Table 2:** Table showing the age distribution of the respondents

S.No	Age	Number of Respondents	Percentage
1	Under 25 years	38	8.5%
2	25-34 years	92	20.4%
3	35-44 years	86	19.1%
4	45-54 years	102	22.7%
5	55-64 years	76	16.9%
6	Beyond 64 years	56	12.4%
	Total	450	100%

The occupations of respondents were business (n=50, 11.1%), self-employed (n=108, 24%), professional (n=108, 24%), students (n=28, 6.2%), teaching staff (n=16, 3.6%), homemakers (n=140, 31.1%), and others (n=148, 32.9%) (Table 3).

**Table 3:** Table showing the occupation of the respondents

S.No	Occupation	Number of Respondents	Percentage
1	Business	50	11.1%
2	Self-employed	108	24%
3	Professional	108	24%
4	Student	28	6.2%
5	Teaching staff	16	3.6%
6	Homemakers	140	31.1%
	Total	450	100%

The education level of respondents was grouped as postgraduate (n=142, 31.6%), graduate (n=216, 48%), schooling (n=86, 19.1%), and no education (n=6, 1.3%) (Table 4).

**Table 4:** Table showing the education level of respondents

S.No	Education Level	Number of Respondents	Percentage
1	Postgraduate	142	31.6%
2	Graduate	216	48%
3	Schooling	86	19.1%
4	No education	6	1.3%
	Total	450	100%

Based on the awareness about the dental services provided by SDC, the respondents were grouped as aware (n=328, 72.9%) and not aware (n=122, 27.1%) (Table 5).

**Table 5:** Table showing the awareness of dental services

S.No	Awareness	Awareness of Dental Services	
		Number of Respondents	Percentage
1	Aware	328	72.9%
2	Not aware	122	27.1%
	Total	450	100%

Based on the visit to SDC, the respondents were grouped as visited (n=428, 95.1%) and did not visit (n=22, 4.9%) (Table 6).

**Table 6:** Table showing the visit history of the respondents SDC: Faculty of Dentistry.

S.No.	Visit to SDC	Number of Respondents	Percentage
1	Visited	428	95.1%
2	Did not visit	22	4.9%
	Total	450	100%

Based on the purpose of the visit to SDC, the respondents were grouped as outpatient (n=126, 28%), diagnostic services (n= 32, 7.1%), visitor (n=270, 60%), and did not visit (n=22, 4.9%) (Table 7).

**Table 7:** Table showing the visit purpose SDC: Faculty of Dentistry.

S.No.	Purpose of Visit to SDC	Number of Respondents	Percentage
1	Outpatient	126	28%
2	Diagnostic services	32	7.1%
3	Visitor	270	60%
4	Did not visit	22	4.9%
	Total	450	100%

Based on the level of satisfaction, the number of respondents was very much satisfied (n=66, 14.7%), satisfied (n=306, 68%), not satisfied (n=46, 10.2%), and not at all satisfied (n=32, 7.1%) (Table 8).

**Table 8:** Table showing the level of satisfaction

S.No.	Satisfaction Level	Number of Respondents	Percentage
1	Very much satisfied	66	14.7%
2	Satisfied	306	68%
3	Not satisfied	46	10.2%
4	Not at all satisfied	32	7.1%
	Total	450	100%

Based on the quality of dental care in comparison to treatment in Tunisia/Egypt, the number of respondents who completely agreed (n=76, 16.9%), who agreed somewhat (n=164, 36.5%), who neither agreed nor disagreed (n=136, 30.2%), who disagreed somewhat (n=36, 8%), and disagreed completely (n=38, 8.4%). Based on the question, SDC will be my preferred hospital in the future: the number of respondents who completely agreed (n=138, 30.7%), who agreed somewhat (n=132, 29.3%), who neither agreed nor disagreed (n=134, 29.8%), who disagreed somewhat (n=28, 6.2%), and disagreed completely (n=18, 4%). Based on the question, in the future, I will recommend SDC to others: the number of respondents who completely agreed (n=148, 32.9%), who agreed somewhat (n=136, 30.2%), who neither agreed nor disagreed (n=118, 26.2%), who disagreed somewhat (n=22, 4.9%), and disagreed completely (n=26, 5.8%) (Table 9).

**Table 9:** Table showing the responses SDC: Faculty of Dentistry.

S.No	Question	Agree Completely		Agree Somewhat		Neither Agree nor Disagree		Disagree Somewhat		Disagree Completely	
		Number	%	Number	%	Number	%	Number	%	Number	%
1	Quality of dental care, treatment in Tunisia/Egypt	76	16.9%	164	36.5%	136	30.2%	36	8%	38	8.4%
2	SDC will be my preferred hospital in the future	138	30.7%	132	29.3%	134	29.8%	28	6.2%	18	4%
3	In the future, I will recommend SDC to others	148	32.9%	136	30.2%	118	26.2%	22	4.9%	26	5.8%

Based on the number of respondents who were aware of caries treatment (n=120), the number of respondents who recalled SDC (n=58, 48.3%) and the percentage of respondents who did not recall SDC (51.7%). Based on the number of respondents who were aware of dental health education (n=302), the number of respondents who recalled SDC (n=172, 56.9%) and the percentage of respondents who did not recall SDC (43.1%). Based on the number of respondents who were aware of oral diagnosis (n=228), the number of respondents who recalled SDC (n=102, 44.7%) and the percentage of respondents who did not recall SDC (55.3%). Based on the number of respondents who were aware of oral surgery (n=152), the number of respondents who recalled SDC (n=124, 81.6%) and the percentage of respondents who did not recall SDC (18.4%). Based on the number of respondents who were aware of tooth replacement (n=142), the number of respondents who recalled SDC (n=132, 92.9%) and the percentage of respondents who did not recall SDC (7.1%). Based on the number of respondents who were aware of gum health (n=144), the number of respondents who recalled SDC (n=100, 69.4%) and the percentage of respondents who did not recall SDC (30.6%) (Table 10).

**Table 10:** Table showing the awareness about specialty services in the Faculty of Dentistry, Sebha SDC: Faculty of Dentistry.

Specialty Services in Dentistry	Total Number of People Aware of the Specialties Among the Respondents (n %)	Total Number of People Who Recalled SDC (Among Those Aware) (n %)	Total Number Who Did Not Recall SDC (n %)
Oral diagnosis	228/450 (50.7%)	102/228 (44.7%)	222/450 (49.3%)
Oral surgery	152/450 (33.8%)	124/152 (81.6%)	298/450 (66.2%)
Tooth replacement	142/450 (31.6%)	132/152 (93%)	308/450 (68.4%)
Gum health	144/450 (32%)	100/144 (69.4%)	306/450 (68%)
Caries treatment	120/450 (26.7%)	58/120 (58.3%)	330/450 (73.3%)
Dental health education	302/450 (67.1%)	172/302 (57%)	148/450 (32.9%)

Only 258 out of 450 (57.3%) responded to the open-ended question. Most comments were positive (n=80, 31%), and many reported the accessibility problem due to distance (n=78, 30.2%). General suggestions were given (n=50, 19.3%) and suggestions to improve in-house services (n=42, 16.2%). Only eight respondents (3.2%) had a bitter experience at SDC (Table 11).

**Table 11:** Table showing comments/views regarding SDC (open-ended question) SDC: Faculty of Dentistry.

S.No	Category	Number of Respondents	Percentage
1	Bitter experience at SDC	8	3.2%
2	The problem of accessibility (distance)	78	30.2%
3	In-house services improvement (suggestions)	42	16.2%
4	Positive opinion	80	31%
5	General suggestions	50	19.3%
	Total	258	100%

## Discussion

A literature search in PubMed using the MESH terms, (("libya"[All Fields]) AND ("dentistry"[All Fields])) AND ("awareness"[All Fields]), showed only four original studies. There were no comparable studies in the Libyan population about awareness of dental hospitals. Jahan et al. [9] performed an awareness study about COVID-19 among non-medical professionals with 137 participants from Southern Libya. Our study had 450 participants from Sebha City in Southern Libya. Elareibi et al. [10] performed an awareness study on sports-related dental emergencies among Libyan sports coaches in Benghazi. Tarhuni et al. [11] performed an awareness study on molar incisor hypomineralization among dentists in Benghazi. Krishnan et al. [12] retrospectively studied indications for the removal of impacted mandibular third molars in Benghazi. To the best of our knowledge, this is the first study to report awareness parameters from Southern Libya.

The study found that while many people were aware of the dental specialties offered by the Sebha Faculty of Dentistry (SDC), a significant portion had not considered SDC for their dental needs. This suggests awareness alone does not translate to preference. Many respondents admitted they had "heard about" SDC or had "no idea" about its facilities. Despite the lack of top-of-mind awareness, a positive takeaway was the high satisfaction rate. Seventy-seven percent of respondents who had used SDC were satisfied with the services. Even when compared to other hospitals, dissatisfaction with SDC was low. Interestingly, when asked about preference, a majority (around 62%) indicated they would choose SDC for future dental needs. Similarly, 62% said they would recommend SDC to others. This suggests positive word-of-mouth plays a significant role in driving preference, even if initial awareness comes from friends and relatives. The study also revealed that friend/relative referrals were the primary source of awareness for SDC.

In Libya, the healthcare system is struggling, with most primary healthcare institutions at higher risk of closure due to shortages of medical staff and supplies, including medications and equipment [12,13]. Furthermore, the situation in Libya has been escalated by the political instability and military conflicts that have resulted in the deterioration of public services, including those for basic needs. All these stressors and enforcements have put extra pressure on civilians' shoulders and have imposed further burdens on them, their families, and the community at large. All the above-mentioned restrictions have the potential to increase the psychosocial burdens of the public in Libya [14]. Patient satisfaction is a measure of the extent to which a patient is content with the healthcare they receive from their healthcare provider. It determines the success of a healthcare facility [15].

Limitations could include uninformed participants, limited geographical distribution of studied samples, interviewer bias, and social desirability bias. Further multi-centric studies across various cities in Libya will provide valuable information about dental services. It will be helpful to plan future policy making.

## Conclusions

This study provides important insights into the improvement of dental facilities in Libya and SDC’s specialty services. The study identified a gap between awareness and preference for SDC. The study also revealed a high satisfaction rate among those who have used SDC's services. The study suggests that SDC holds an edge in technical expertise. By addressing the awareness gap and capitalizing on its strengths, SDC can solidify its position as the preferred choice for dental care in Sebha.

## Appendices

Questionnaire

I am Sathya Sethuraman/Syed Wali Peeran/Aesa Alzharoug Jaber/Karthikeyan Ramalingam. 

We are conducting a survey on Awareness about the dental health facilities in Sebha along with the Faculty of Dentistry, Sebha.

I _________________________________________ agree to take part in this study, conducted by the Faculty of Dentistry, Sebha. As an informed participant in this study, I understand that my participation in this study is voluntary and I may cease to take part in this study at any time, without any penalty.

There are no risks involved in the participation of this study. All my questions about the study have been satisfactorily answered. I have read and understood the above and also I declare that I permit the above-mentioned individual/ organization/ hospital/ clinic/ laboratory to use my details for scientific purposes (education, research, publication) and I give consent to participate in this study.

Participant’s Signature:__________________________________ Date:__________

Researcher’s Signature:__________________________________ Date:__________

The details are asked purely for research purposes and to improve the health services to the public. Kindly spend a few minutes to fill this form. Please try to answer all the questions.

1) To help us analyze this survey please give us the following details:

a) Gender 🖵  Male         🖵 Female

b) Your approximate age in years? 

🖵 under 25       🖵 25 - 34        🖵 35 - 44 🖵 45 - 54     🖵 55 - 64 

c) Your Occupation 

🖵 Business 🖵 Self-employed 🖵  Professional                                                     

🖵  Student           🖵 Teaching         🖵  Executive / Employed

🖵 Homemaker Others

d) e) Educational Qualification?

🖵 Post Graduate                   🖵  Graduate

🖵 Completed my schooling      🖵  Did not undergo education                               

🖵 others, ____________

2.    Which specialty comes to your mind first when it comes to the following? 

S.No

Specialties

SDC

1

Oral diagnosis

🖵

2

Oral surgery

🖵

3

Tooth replacement

🖵

4

Gum health

🖵

5

Caries treatment

🖵

6

Dental health education

🖵

3) Are you aware of the following facilities/Services provided by SDC?

S.No

Facilities/Services Provided

Aware(1)

Not aware (2)

1.

Oral Diagnosis

🖵

🖵

2

Oral surgery

🖵

🖵

3

Tooth replacement

🖵

🖵

4

Gum health

🖵

🖵

5

Caries treatment

🖵

🖵

6

Dental health Education

🖵

🖵

4) If your answer to the above question is “I know it” or “I have heard about it”, how did you come to know about the facilities?

🖵 Through friends / Relatives  🖵 Family Doctor 🖵 Patient references 🖵 Advertisements in newspapers       🖵 because SDC is very popular 🖵 through people working in SDC    🖵 others _________________________

5 There are a few statements written below, for each one we would like you to tell whether you agree or disagree with it. (READ THE STATEMENTS BELOW)

S.No

Statement

Agree

Completely 

Agree

Somewhat

Neither

Agree nor

Disagree

Disagree

Somewhat

Disagree

Completely

1

The quality of dental care provided by SDC can be compared with international standards 

🖵

🖵

🖵

🖵

🖵

2

SDC will be my most preferred hospital when it comes to dental health

🖵

🖵

🖵

🖵

🖵

3

In future, I will recommend SDC to others

🖵

🖵

🖵

🖵

🖵

6. i) Have you been to  SDC? 

🖵 Yes 🖵 No

ii) If yes, for what purpose?

🖵 As an outpatient   🖵 for diagnostic services 🖵 Came as a visitor                       🖵Others, Specify ____________________________________________________

iii) What was your satisfaction level at SDC

🖵  Very Satisfied 🖵  Satisfied 🖵  Not Satisfied 🖵  Not at all Satisfied

7) Your comments, suggestions, and views regarding SDC: ______________________________________________________________________________________________________________________________________________________________________________________________________________________________________________________________________________________________________________________________________________________________________________________________________________________________________________

Thank you for your assistance and cooperation in the survey.
